# Supplementary material for: Analysis of mutational and proteomic heterogeneity of gastric cancer suggests an effective pipeline to monitor post-treatment tumor burden using circulating tumor DNA
Source: PLoS One. 2020 Oct 7;15(10):e0239966. doi: 10.1371/journal.pone.0239966 (PMC7540850; doi:10.1371/journal.pone.0239966)
Supplement: S1 Table — (DOCX) [file pone.0239966.s012.docx]

**S1 Table. Patient characteristics**

| ID | Age | Sex | size (mm) | Pathological findings | | | | Adjuvant treatment | Time to last follow-up (days)* | Clinical recurrence (days)* | Recurrence site |
| --- | --- | --- | --- | --- | --- | --- | --- | --- | --- | --- | --- |
|  |  |  |  | T^a^ | N^a^ | pStage^a^ | Histology^b^ |  |  |  |  |
| GC1 | 66 | Male | 50 x 40 | pT3 | pN2 | IIIA | Intestinal | No | 919 | No | No |
| GC2 | 79 | Male | 105 x 95 | pT4a | pN3b | IIIC | Diffuse | No | 176 | Yes (176) | Peritoneal dissemination |
| GC3 | 70 | Female | 60 x 50 | pT3 | pN2 | IIIA | Intestinal | S-1  one year | 1005 | No | No |
| GC4 | 87 | Female | 22 x 20 | pT1 | pN0 | IA | Intestinal | No | 909 | No | No |
| GC6 | 63 | Female | 20 x 21 | pT3 | pN0 | IIA | Diffuse | No | 977 | No | No |
| GC7 | 68 | Female | 75 x 60 | pT4a | pN2 | IIIB | Diffuse | S-1  one year | 921 | No | No |
| GC8 | 83 | Female | 35 x 25 | pT4a | pN0 | IIB | Intestinal | No | 970 | No | No |
| GC12 | 72 | Male | 75 x 65 | pT4b | pN3a | IIIC | Intestinal | CapeOX  three months | 972 | Yes (544) | Peritoneal dissemination |
| GC13 | 60 | Female | 150 x 140 | pT3 | pN0 | IIB | Intestinal | S-1  one year | 902 | No | No |
| GC14 | 62 | Male | 50 x 35 | pT1 | pN1 | IIB | Intestinal | No | 739 | No | No |

Abbreviations: CapeOX., Capecitabine plus Oxaliplatin.

^a^TNM Classification of Malignant Tumors, 8th Edition. ^b^Lauren classification. *After surgery.

S-1, an oral fluoropyrimidine. Capecitabine, an oral fluoropyrimidine. Oxaliplatin, a third-generation platinum complex
