# Supplementary material for: Analysis of mutational and proteomic heterogeneity of gastric cancer suggests an effective pipeline to monitor post-treatment tumor burden using circulating tumor DNA
Source: PLoS One. 2020 Oct 7;15(10):e0239966. doi: 10.1371/journal.pone.0239966 (PMC7540850; doi:10.1371/journal.pone.0239966)
Supplement: S2 Table — (DOCX) [file pone.0239966.s013.docx]

**S2 Table. Cellularity of multi-region samples**

| ID | Region | Cellularity  (%) |
| --- | --- | --- |
| GC1 | 1 | >80 |
|  | 2 | >80 |
|  | 3 | 70 |
| GC2 | 1 | 70 |
|  | 2 | 75 |
|  | 3 | 75 |
| GC3 | 1 | 70 |
|  | 2 | 40 |
|  | 3 | 50 |
| GC4 | 1 | 50 |
|  | 2 | 50 |
|  | 3 | 30 |
| GC6 | 1 | 40 |
|  | 2 | 50 |
|  | 3 | 60 |
| GC7 | 1 | 75 |
|  | 2 | 35 |
|  | 3 | 65 |
| GC8 | 1 | 80 |
|  | 2 | 30 |
|  | 3 | 70 |
| GC12 | 1 | 90 |
|  | 2 | 85 |
|  | 3 | 75 |
| GC13 | 1 | 90 |
|  | 2 | 90 |
|  | 3 | 90 |
| GC14 | 1 | 40 |
|  | 2 | 70 |
|  | 3 | 75 |
