# Supplementary material for: Analysis of mutational and proteomic heterogeneity of gastric cancer suggests an effective pipeline to monitor post-treatment tumor burden using circulating tumor DNA
Source: PLoS One. 2020 Oct 7;15(10):e0239966. doi: 10.1371/journal.pone.0239966 (PMC7540850; doi:10.1371/journal.pone.0239966)
Supplement: S3 Table — (DOCX) [file pone.0239966.s014.docx]

**S3 Table. Tumor-unique mutations present in 10 cases**

| ID | Gene | Location | Nucleotide mutation | Amino acid change | Founder/ non-founder mutation | Region 1 | | | Region 2 | | | Region 3 | | |
| --- | --- | --- | --- | --- | --- | --- | --- | --- | --- | --- | --- | --- | --- | --- |
|  |  |  |  |  |  | Cov | Var.cov | VAF  (%) | Cov | Var.cov | VAF (%) | Cov | Var.cov | VAF (%) |
| GC1 | *TP53* | chr17:7577538 | c.743C>T | R248Q | Founder | 221 | 164 | 74.21 | 243 | 69 | 28.4 | 206 | 116 | 56.31 |
| GC1 | *SLCO1B1* | chr12:21329707 | C>T | Splice site | Non-founder | 304 | 21 | 6.91 | － | － | － | － | － | － |
| GC2 | *RB1* | chr13:49050980 | G>A | Splice site | Founder | 528 | 200 | 37.88 | 430 | 139 | 32.33 | 356 | 114 | 32.02 |
| GC3 | *TP53* | chr17:7577517 | c.586G>A | R196* | Founder | 592 | 137 | 23.14 | 718 | 106 | 14.76 | 852 | 153 | 17.96 |
| GC4 | *ERBB2* | chr17:37866444 | c.749C>T | S250F | Non-founder | 2007 | 147 | 7.32 | － | － | － | 1470 | 76 | 5.17 |
| GC6 | *CTNNB1* | chr3:41266682 | c.479T>C | L160P | Founder | 824 | 62 | 7.52 | 831 | 51 | 3.14 | 822 | 95 | 11.56 |
| GC6 | *ERBB4* | chr2:212248507 | c.3730G>T | Q1244K | Non-founder | － | － | － | 2130 | 126 | 5.92 | 2111 | 242 | 11.46 |
| GC6 | *IKZF1* | chr7:50468024 | c.1259C>T | P420L | Non-founder | － | － | － | － | － | － | 929 | 98 | 10.55 |
| GC7 | *TP53* | chr17:7577517 | c.764A>G | I255T | Founder | 177 | 62 | 35.03 | 197 | 131 | 36.5 | 218 | 41 | 18.81 |
| GC7 | *MAP3K1* | chr5:56177090 | c.2360T>C | V787A | Founder | 656 | 141 | 21.49 | 888 | 268 | 30.18 | 965 | 90 | 9.33 |
| GC7 | *AKT3* | chr1:243736256 | c.791G>A | S264F | Founder | 675 | 113 | 16.74 | 872 | 201 | 23.05 | 1044 | 76 | 7.28 |
| GC7 | *PIK3CA* | chr3:178936091 | c.1633G>A | E545K | Non-founder | 56 | 5 | 8.93 | 80 | 17 | 21.25 | － | － | － |
| GC7 | *SMAD4* | chr18:48584560 | c.733C>T | Q245* | Non-founder | 334 | 55 | 16.47 | － | － | － | － | － | － |
| GC8 | *PIK3CA* | chr3:178952085 | c.3140A>G | H1047R | Non-founder | 566 | 57 | 10.07 | 626 | 83 | 13.26 | － | － | － |
| GC8 | *TP53* | chr17:7579313 | c.374G>A | T125M | Non-founder | 737 | 81 | 10.99 | － | － | － | － | － | － |
| GC8 | *ERBB3* | chr12:56478786 | c.242G>A | R81Q | Non-founder | － | － | － | 1236 | 136 | 11 | － | － | － |
| GC8 | *KIT* | chr4:55604606 | c.2802C>G | N934K | Non-founder | 1135 | 136 | 11.98 | － | － | － | － | － | － |
| GC12 | *TP53* | chr17:7578190 | c.659T>C | Y220C | Founder | 152 | 90 | 59.21 | 155 | 94 | 60.65 | 204 | 88 | 43.14 |
| GC12 | *MAP2K4* | chr17:11998898 | c.122C>T | S41L | Non-founder | 183 | 20 | 10.93 | － | － | － | － | － | － |
| GC12 | *LAMA2* | chr6:129468275 | C>T | Intron variant | Non-founder | － | － | － | 198 | 71 | 35.86 | － | － | － |
| GC13 | *IKZF1* | chr7:50467828 | c.1063G>A | A355T | Founder | 377 | 129 | 34.22 | 381 | 60 | 15.75 | 386 | 98 | 25.39 |
| GC13 | *ATM* | chr11:108236203 | c.9139C>T | R3047* | Non-founder | 1133 | 385 | 33.98 | － | － | － | 863 | 199 | 23.06 |
| GC13 | *PIK3CA* | chr3:178952084 | c.3139C>T | H1047Y | Non-founder | 570 | 172 | 30.18 | － | － | － | 398 | 114 | 28.64 |
| GC13 | *ERBB4* | chr2:212248612-212248613 | c.3654_3655insT | Frameshift | Non-founder | 1623 | 460 | 28.34 | － | － | － | － | － | － |
| GC14 | *TP53* | chr17:7578406 | c.524C>T | R175H | Founder | 791 | 293 | 37.04 | 855 | 360 | 42.11 | 818 | 159 | 19.44 |
| GC14 | *SMAD4* | chr18:48593397 | c.413T>A | I138K | Founder | 421 | 157 | 37.29 | 471 | 190 | 40.34 | 432 | 53 | 12.77 |

Abbreviations: cov., coverage; var. cov., variant coverage, VAF; variant allele frequency.
