# Supplementary material for: Analysis of mutational and proteomic heterogeneity of gastric cancer suggests an effective pipeline to monitor post-treatment tumor burden using circulating tumor DNA
Source: PLoS One. 2020 Oct 7;15(10):e0239966. doi: 10.1371/journal.pone.0239966 (PMC7540850; doi:10.1371/journal.pone.0239966)
Supplement: S4 Table — (DOCX) [file pone.0239966.s015.docx]

**S4 Table. Digital PCR analysis using mutation-specific primer/probe sets**

| ID | Gene | Location | Nucleotide mutation | Amino acid change | Founder/ non-founder mutation | Validation of primer/probe sets by dPCR | | | | | | | | | dPCR for preoperative plasma | | |
| --- | --- | --- | --- | --- | --- | --- | --- | --- | --- | --- | --- | --- | --- | --- | --- | --- | --- |
|  |  |  |  |  |  | Region 1 | | | Region 2 | | | Region 3 | | |  |  |  |
|  |  |  |  |  |  | Total allele  count | Mutant. allele  count | VAF (%) | Total allele  count | Mutant. allele  count | VAF (%) | Total allele  count | Mutant. allele  count | VAF (%) | Total allele  count | Mutant. allele  count | VAF (%) |
| GC1 | *TP53* | chr17:7577538 | c.743C>T | R248Q | Founder | 8033 | 6137 | 76.40 | - | - | - | - | - | - | 830 | 11 | 1.33 |
| GC1 | *SLCO1B1* | chr12:21329707 | C>T | Splice site | Non-founder | 9979 | 572 | 5.73 | 5947 | 2 | 0.03 | 12881 | 2 | 0.02 | 302 | 0 | 0 |
| GC2 | *RB1* | chr13:49050980 | G>A | Splice site | Founder | 4738 | 1880 | 39.68 | - | - | - | - | - | - | 616 | 0 | 0 |
| GC3 | *TP53* | chr17:7577517 | c.586G>A | R196* | Founder | 2772 | 694 | 25.04 | - | - | - | - | - | - | 1016 | 0 | 0 |
| GC4 | *ERBB2* | chr17:37866444 | c.749C>T | S250F | Non-founder | 4625 | 330 | 7.14 | 3866 | 126 | 3.26 | 7483 | 288 | 3.85 | 1626 | 0 | 0 |
| GC6 | *CTNNB1* | chr3:41266682 | c.479T>C | L160P | Founder | - | - | - | - | - | - | 4585 | 546 | 11.91 | 521 | 0 | 0 |
| GC6 | *ERBB4* | chr2:212248507 | c.3730G>T | Q1244K | Non-founder | 3587 | 7 | 0.20 | 7693 | 274 | 3.56 | 4479 | 492 | 10.98 | 550 | 0 | 0 |
| GC6 | *IKZF1* | chr7:50468024 | c.1259C>T | P420L | Non-founder | 3343 | 182 | 5.44 | 6138 | 195 | 3.18 | 3014 | 372 | 12.34 | 689 | 0 | 0 |
| GC7 | *TP53* | chr17:7577517 | c.764A>G | I255T | Founder | - | - | - | 2777 | 1238 | 44.58 | - | - | - | 810 | 0 | 0 |
| GC7 | *MAP3K1* | chr5:56177090 | c.2360T>C | V787A | Founder | - | - | - | 2820 | 589 | 20.89 | - | - | - | 754 | 0 | 0 |
| GC7 | *AKT3* | chr1:243736256 | c.791G>A | S264F | Founder | - | - | - | 3302 | 683 | 20.68 | - | - | - | 660 | 0 | 0 |
| GC7 | *PIK3CA* | chr3:178936091 | c.1633G>A | E545K | Non-founder | 4604 | 954 | 20.72 | 5564 | 1686 | 29.77 | 3978 | 160 | 4.02 | 737 | 0 | 0 |
| GC7 | *SMAD4* | chr18:48584560 | c.733C>T | Q245* | Non-founder | 2315 | 364 | 15.72 | 2572 | 3 | 0.12 | 2283 | 3 | 0.13 | 572 | 0 | 0 |
| GC8 | *PIK3CA* | chr3:178952085 | c.3140A>G | H1047R | Non-founder | 2996 | 276 | 9.21 | 4148 | 473 | 11.4 | 3815 | 77 | 2.02 | 222 | 0 | 0 |
| GC8 | *TP53* | chr17:7579313 | c.374G>A | T125M | Non-founder | 4454 | 414 | 9.30 | 2801 | 4 | 0.14 | 3119 | 2 | 0.06 | 207 | 0 | 0 |
| GC8 | *ERBB3* | chr12:56478786 | c.242G>A | R81Q | Non-founder | 2851 | 0 | 0 | 3144 | 349 | 11.10 | 5286 | 97 | 1.84 | 281 | 0 | 0 |
| GC8 | *KIT* | chr4:55604606 | c.2802C>G | N934K | Non-founder | 3945 | 308 | 7.81 | 3880 | 4 | 0.10 | 4460 | 98 | 2.20 | 182 | 0 | 0 |
| GC12 | *TP53* | chr17:7578190 | c.659T>C | Y220C | Founder | - | - | - | 673 | 425 | 63.15 | - | - | - | 1767 | 22 | 1.25 |
| GC12 | *MAP2K4* | chr17:11998898 | c.122C>T | S41L | Non-founder | 2362 | 314 | 13.29 | 1337 | 4 | 0.30 | 2505 | 1 | 0.04 | 3108 | 1 | 0.03 |
| GC12 | *LAMA2* | chr6:129513973 | C>T | Intron variant | Non-founder | 1882 | 573 | 30.45 | 1194 | 461 | 38.61 | 1162 | 318 | 27.37 | 2027 | 9 | 0.44 |
| GC13 | *IKZF1* | chr7:50467828 | c.1063G>A | A355T | Founder | 1470 | 665 | 45.24 | - | - | - | - | - | - | 572 | 4 | 0.70 |
| GC13 | *ATM* | chr11:108236203 | c.9139C>T | R3047* | Non-founder | 3370 | 1733 | 33.96 | 3122 | 52 | 1.67 | 2286 | 668 | 29.22 | 685 | 3 | 0.44 |
| GC13 | *PIK3CA* | chr3:178952084 | c.3139C>T | H1047Y | Non-founder | 3769 | 1291 | 34.25 | 3355 | 56 | 1.67 | 2910 | 537 | 18.45 | 649 | 2 | 0.31 |
| GC13 | *ERBB4* | chr2:212248612-212248613 | c.3654_3655insT | frameshift | Non-founder | 2239 | 1011 | 45.15 | 1714 | 27 | 1.58 | 685 | 2 | 0.29 | 461 | 1 | 0.22 |
| GC14 | *TP53* | chr17:7578406 | c.524C>T | R175H | Founder | - | - | - | 4636 | 1836 | 39.60 | - | - | - | 2073 | 0 | 0 |
| GC14 | *SMAD4* | chr18:48593397 | c.413T>A | I138K | Founder | - | - | - | 2890 | 1155 | 39.97 | - | - | - | 1526 | 0 | 0 |

Abbreviations: dPCR., digital PCR, VAF; variant allele frequency.
