# Supplementary material for: Analysis of mutational and proteomic heterogeneity of gastric cancer suggests an effective pipeline to monitor post-treatment tumor burden using circulating tumor DNA
Source: PLoS One. 2020 Oct 7;15(10):e0239966. doi: 10.1371/journal.pone.0239966 (PMC7540850; doi:10.1371/journal.pone.0239966)
Supplement: Attachment — Submitted filename: Response_to_Reviewers.docx [file pone.0239966.s016.docx]

Response to Reviewers:

**Reviewer #1:**

**The study is multifaceted so the overarching theme is unclear at times. The investigators analyzed 10 gastric cancers by performing targeted sequencing and proteomic analysis of three regions of each tumor. They identified several tumor specific mutations and protein profiles. Mutations were then analyzed in circulating tumor DNA to determine whether they could be used as biomarkers for treatment response. The data were strong and mostly support the conclusions that were drawn. However, the authors need to address several concerns.**

>Thank you very much for your constructive comments. In particular, we are encouraged by the words, “***The data were strong and mostly support the conclusions that were drawn***”. We believe that we have now fully responded to the points raised.

**1) The Abstract is very difficult to follow as compared to the Author Summary which was well written. I suggest that the Abstract be written so the reader can more easily follow the logic of the study.**
>The Abstract has been largely rewritten based on the Author Summary. Thank you for your suggestion.

*Circulating tumor DNA (ctDNA) is released from tumor cells into blood in advanced cancer patients. Although gene mutations in individual tumors can be diverse and heterogenous, ctDNA has the potential to provide comprehensive biomarker information. Here, we performed multi-region sampling (three sites) per resected specimen from 10 gastric cancer patients followed by targeted sequencing and proteomic profiling using reverse-phase protein arrays. A total of 126 non-synonymous mutations were identified from 30 samples from 10 tumors. Of these, 16 (12.7%) were present in all three regions and were designated as founder mutations. Variant allele frequencies (VAFs) of founder mutations were significantly higher than those of non-founder mutations. Phylogenetic analysis also demonstrated a good concordance between founder and truncal mutations, defined as mutations shared by all simulated clones at the trunk of the tumor phylogenetic tree. These findings led us to prioritize founder mutations for quantitative ctDNA monitoring by digital PCR with individually-designed primer/probe sets. In preoperative plasma, the average ctDNA VAF of founder mutations was significantly higher than that of non-founder mutations (p = 0.039). Proteomic heterogeneity was present across the tumor regions both within and between patients independent of mutational status. Our results suggest that, in practice, mutations having high VAF identified without multi-regional sequencing may be immediately useful for quantitative ctDNA monitoring but do not provide sufficient information to predict the proteomic composition of tumors.*

**2) The term founder and non-founder mutations is used to describe whether a mutation is present in all or some of the regions of a tumor. This terminology is fine. However, others have used the terminology of public versus private. Public is present in all regions, whereas private is present in only some.**
>Thank you for pointing this out. In fact, we understand the terminology of public versus private, which are fully matched conceptually to what we had intended to define founder and non-founder, respectively (Gerlinger et al, ***NEJM***, 2012; Sottoriva et al, ***Nat Genet***, 2015). The definition of founder mutations has also been termed clonal, ubiquitous, and shared (Trajlic et al, ***BBA***, 2015; Saito et al, ***Nat Commun***, 2018). However, of note, these terms are based on binarized data (i.e., the presence or absence of mutations) whereas the term “truncal”, which is the term used for the results presented in the phylogenetic tree, is based on simulations using VAF data as continuous variables. Therefore, we differentiated between the concept of founder, public, clonal, ubiquitous, and shared mutations from truncal mutations. We have added a list of references to describe equivalent observations as our definition for founder and non-founder mutations. Please see Page 12, Line 228.

“*The concept of “founder and non-founder” mutations has also been termed “public and private” for clonal and subclonal mutations in a tumor　[23, 24].* *These terms are based on binarized data (i.e., the presence or absence of mutations), whereas the term “truncal”, which is used for the results presented in the phylogenetic tree, is based on simulations using VAF data as continuous variables.*”

**3) The authors define non-founding as mutations based on Next Gen as being present in only SOME regions. Yet, digital PCR revealed that many non-founding mutations were actually present in all regions. Would it be better to reclassify such "non-founding" as actually "founding"? Should you use three classes - public, consistent VAF; public, low or inconsistent VAF, and private.
From the Abstract, "93% of non-founder** **mutations were found in all three regions". Aren't these non-founders actually founders by you definition.**
>Thank you very much for raising this important point. We validated only 15 of the 110 non-founder mutations for which dPCR primer/probe sets were designed. Therefore, we have not been able to fully categorize our NGS data as you suggested at present. We agree with your point and indeed had an impression that the “true” non-founder mutations may be fewer than we have seen if we use a highly sensitive method, such as dPCR. The sentence from the original abstract “*93% of non-founder mutations were found in all three regions*” can only be applied for “Among those validated” samples (n=15). Hence, it may not be generalized with the present data. We have deleted the sentence in the updated abstract. The following sentence has been added on Page 16, Line 307.

“*An exhaustive validation of NGS-based multiregional sequencing using highly sensitive dPCR will be warranted for establishing true founder mutations.*”

**4)-1 The authors did a good job of presenting the data. Presumably the phylogenetic trees force that notion that each tumor is derived from a single rogue cell.**

>Thank you very much for your nice compliment. As you presumed, the pre-simulation assumption for phylogenetic trees coded by a modified Canopy algorithm is that all cancer clones ultimately originate from a normal cell. In addition to the absence of whole genome sequencing for all regional samples, we must admit that there is a limit of phylogenetic tree simulation due to the nature of the Canopy algorithm stated in the original article, “*we do not directly observe the clones; instead the samples we sequence are mixtures*” (Jiang et al, ***PNAS***, 2016). A relevant sentence was inserted in the Discussion section on Page 21, Line 429.

“*The exhaustive assessment of multiregional sequencing may alter the initial condition of the phylogenetic simulation.*”

**4)-2 Is there any evidence that some of these cancers could actually be derived from multiple different rogue cells?**

In response to the interesting point that the reviewer raised, in fact, we observed some tumors that do not have a truncal mutation, including GC2, GC6, GC8 (hypermutator), GC13 (hypermutator), and GC14, while most of them had founder mutations. These observations may be because the tumor was derived from multiple different transformed cells. However, the observations were not exhaustive to fully conclude that there may be multiple origins of a tumor. Rather, it may be likely due to technical limitations from the definition of founder (based on binary variables) and truncal (based on continuous variables) mutations.

**4)-3 Figure 1 demonstrates that some genes are mutant in all three regions of a tumor but is the mutation always the same mutation. Please state explicitly in legend.**
>Thank you for the suggestion. Three genes include different mutations in at least one of three regions of a tumor: *PIK3CA*, *INPP4B,* and *ABCC2* of GC13. *INPP4B* and *ABCC2* were not marked with arrows as notable mutations in Figure 1 in the original version. Primer/probe sets for digital PCR were not designed for these two mutations. We added the following sentence to the Figure 1 and Figure S2 legends as well as arrows indicating *INPP4B* and *ABCC2* of GC13 in Figure 1. However, please note that the GC13 is considered to be a hypermutator, which may not be representative of tumor clonal evolution.

“*Mutations seen in more than one region in a tumor may not always represent the same mutation.* *The following genes/case include different mutations in at least one of three regions of a tumor: PIK3CA/GC13, INPP4B/GC13, and ABCC2/GC13.*”

**5)-1 Is it apparent that mutation profile would correlate with protein heterogeneity. can other studies in which this correlation is tested be cited.**

>Thank you for pointing this out. We unintentionally missed the necessary citations. We have now cited previous proteogenomic studies that have suggested that protein abundance cannot be reliably predicted from DNA-level measurements (Zhang et al, ***Nature***, 2014; Mertins et al, ***Nature***, 2016; Mun et al, ***Cancer Cell***, 2018). We have inserted a relevant paragraph and citations in the Results subsection, “Protein level prediction by gene mutation” on Page 17, Line 337.

“*Previous reports have suggested that protein abundance measured by mass spectrometry cannot be reliably predicted from DNA measurements [27-29]. In the present study, ……..*”

**5)-2 It seems with targeted sequencing and limited protein analysis that finding any correlation would be quite a long shot unless very, very significant.**
>We agree with the reviewer’s comment. It is a long shot. Indeed, there has been a very limited number of good antibodies for true target detection, such as phosphorylated targets. Therefore, it is difficult to completely capture the entire picture of signal transduction. In addition, the prevalence of mutations per gene is low in general. If the prevalence of mutations is 5% in a population, then it requires at least 100 samples to identify 5 samples with mutations in the gene. In the present study, we compared 12 gene-protein relationships in 42 gene-protein pairs from a 151 gene sequencing panel and 294 RPPA protein panel with a minimum group sample number of three. Although the number of cases was small, the multiregional sampling allowed us to compare the gene-protein relationships in 40-region samples, of which each sample holds gene-protein information. Again, we must admit the comparison is a long shot. However, for instance, we demonstrated that there was an expected result in the present platform for *TP53* and p53. Recognizing the limitation from the present study, we are now expanding the number of more than 200 Pan-Cancer sample collection with our university hospital-wide clinical study (MORIOKA study; jRCT1020190017) for network-level gene-protein relationship analyses instead of that of a target protein-level.

**6) Does the ctDNA markers perform better than current markers in assessing treat response and disease free survival? Please provide a statistical analysis.**

>A long-term prognosis, such as disease-free survival, was not included in the evaluation objects at the time of conceptualization in this observational study. Therefore, except for case presentation, we are still in the process of accumulating longitudinal data of ctDNA monitoring of gastric cancer as a part of our Pan-Cancer project described above (MORIOKA study; jRCT1020190017). In the present study, ctDNA was detected in only three of 10 cases, and therefore we felt that the dataset was too small to compare the clinical validity of ctDNA with currently-used biomarkers, such as CEA. Our separate study of ctDNA monitoring of esophageal squamous cell cancer (ESCC) by Iwaya et al, demonstrated that 91% of ESCC patients benefited from ctDNA monitoring with the clinical validity of an individualized biomarker. The clinical validity was evaluated in terms of early relapse prediction, treatment efficacy evaluation, and non-relapse confirmation, while the clinical validity of conventional serum markers for SCC, CYFRA, and CEA showed much less impact than that of ctDNA, particularly in early relapse prediction (under review; doi: https://doi.org/10.1101/2020.05.01.20087106). In terms of actual survival benefit correlation with ctDNA monitoring, we found that the ESCC group whose ctDNA decreased after treatment had a hazards ratio of 0.1 compared to the group whose ctDNA was sustained. In the current manuscript, we further discuss the clinical utility of ctDNA monitoring in comparison with current biomarkers in terms of treatment response on Page 22, Line 451.

“*It seems that not all gastric cancer cases can be monitored by ctDNA alone. Further observational studies are warranted to determine the clinical validity of ctDNA in gastric cancer diagnosis, in comparison with current modalities, such as serum markers and imaging studies.*”

**Reviewer #2: Good report, will likely significantly advance the use of circulating tumor DNA. Authors are to be commended for careful IRB record notations and excellent supporting online material file, data transparency records.**

>Thank you very much for your comment. We are excited to proceed to our next Pan-Cancer study as well as intervention research based on our current data.
